# Retraction Note: Calycosin-7-O-β-D-glucoside promotes oxidative stress-induced cytoskeleton reorganization through integrin-linked kinase signaling pathway in vascular endothelial cells

**DOI:** 10.1186/s12906-022-03817-5

**Published:** 2022-12-10

**Authors:** Yue-Hua Jiang, Wei Sun, Wei Li, Hong-Zhen Hu, Le Zhou, Hui-Hui Jiang, Jing-Xin Xu

**Affiliations:** 1 Affiliated Hospital of Shandong University of Traditional Chinese Medicine, West Wenhua road #42, Jinan, Shandong 250011 PR China; 2 Jiangsu Province Hospital of Traditional Chinese Medicine, Nanjing, 210029 China

**Retraction Note: BMC Complement Altern Med 15, 315 (2015)**

**https://doi.org/10.1186/s12906-015-0839-5**

The Editor has retracted this article because of significant concerns regarding a number of Figures presented in this work, which question the integrity of the data. Specifically:

- Image overlap has been found in Figure 3A between images with different data labels: Normal HUVECs and LPS+valsartan; as well as LPS+Calycosin and LPS+valsartan
- It appears that Figure 7c B-actin blot has unexpected similarity to Figure 7A actin blot, although this image has been flipped and edited.

In addition, there are inconsistencies between Figure 7 and the underlying data. The Editor therefore no longer has confidence in the integrity of the data in this article.

Author Yue-Hua Jiang agrees to this retraction. Corresponding author Wei Li has stated on behalf of all co-authors that they agree to this retraction.

